# VLDL/LDL acts as a drug carrier and regulates the transport and metabolism of drugs in the body

**DOI:** 10.1038/s41598-017-00685-9

**Published:** 2017-04-04

**Authors:** Hideaki Yamamoto, Tappei Takada, Yoshihide Yamanashi, Masatsune Ogura, Yusuke Masuo, Mariko Harada-Shiba, Hiroshi Suzuki

**Affiliations:** 1 Department of Pharmacy, The University of Tokyo Hospital, Faculty of Medicine, The University of Tokyo, Tokyo, 113-8655 Japan; 2 Department of Molecular Innovation in Lipidology, National Cerebral and Cardiovascular Center Research Institute, Osaka, 565-8565 Japan

## Abstract

Only free drugs have been believed to be carried into tissues through active or passive transport. However, considering that lipoproteins function as carriers of serum lipids such as cholesterol and triglycerides, we hypothesized that lipoproteins can associate with certain drugs and mediate their transport into tissues in lipid-associated form. Here, *in vitro* and *in vivo* studies with low density lipoprotein receptor (LDLR)-overexpressing or -knockdown cells and wild-type or LDLR-mutant mice were used to show the association of various drugs with lipoproteins and the uptake of lipoprotein-associated drugs through a lipoprotein receptor-mediated process. In clinical studies, investigation of the effect of lipoprotein apheresis on serum drug concentrations in patients with familial hypercholesterolemia demonstrated that lipoprotein-mediated drug transport occurs in humans as well as in mice. These findings represent a new concept regarding the transport and metabolism of drugs in the body and suggest that the role of lipoprotein-mediated drug transport should be considered when developing effective and safe pharmacotherapies.

## Introduction

Drugs present in blood are generally classified into two forms, a protein-bound form and an unbound free form. In pharmacokinetics, it is generally believed that only free drugs can transfer to tissues^1, 2^. However, serum concentrations of free drugs are not always associated with predictable pharmacokinetic outcomes or pharmacological effects^3–5^. To address such unpredictable drug behaviors, we focused on possible interactions between drugs and lipoproteins that are taken up by tissues via receptor-mediated endocytosis.

The main physiological role of lipoproteins is to transport lipids such as cholesterol and triglycerides in the hydrophilic environment of the circulatory system. Lipoproteins are usually classified according to their density as chylomicron, very low density lipoprotein (VLDL), low density lipoprotein (LDL) and high density lipoprotein (HDL). Studies show that lipoproteins can deliver certain lipophilic compounds such as fat-soluble vitamins and endocrine-disrupting substances, in addition to cholesterol and triglycerides^6–8^. Therfore, we hypothesized that drugs may also associate with lipoproteins and this association may affect the behaviors (transport and metabolism) of drugs through receptor-mediated uptake of lipoprotein-associated drugs into tissues (Supplementary Fig. S1).

The present study reveals an important role of lipoproteins in the distribution of drugs in the body. *In vivo* studies in mice showed that various drugs associate with lipoproteins, and that changes in VLDL and/or LDL (VLDL/LDL) metabolism influence the behavior of drugs. *In vitro* studies using LDL receptor (LDLR)-overexpressing and -knockdown cells demonstrated that the behavior of VLDL/LDL-associated drugs can be regulated by LDLR, which was consistent with *in vivo* observations that the behavior of VLDL/LDL-associated drugs was altered in mutant mice lacking functional LDLR (LDLR-MT). Our findings in mouse models were confirmed in clinical studies showing that, similar to lipids, VLDL/LDL-associated drugs were dramatically eliminated from the body by lipoprotein apheresis, a blood purification therapy that selectively removes VLDL/LDL particles from the bloodstream. These results demonstrated the importance of VLDL/LDL-mediated drug transport, which can influence transport, metabolism, and the efficacy of drugs in humans.

## Results

### Association of various drugs with lipoproteins

**Figure 1:**
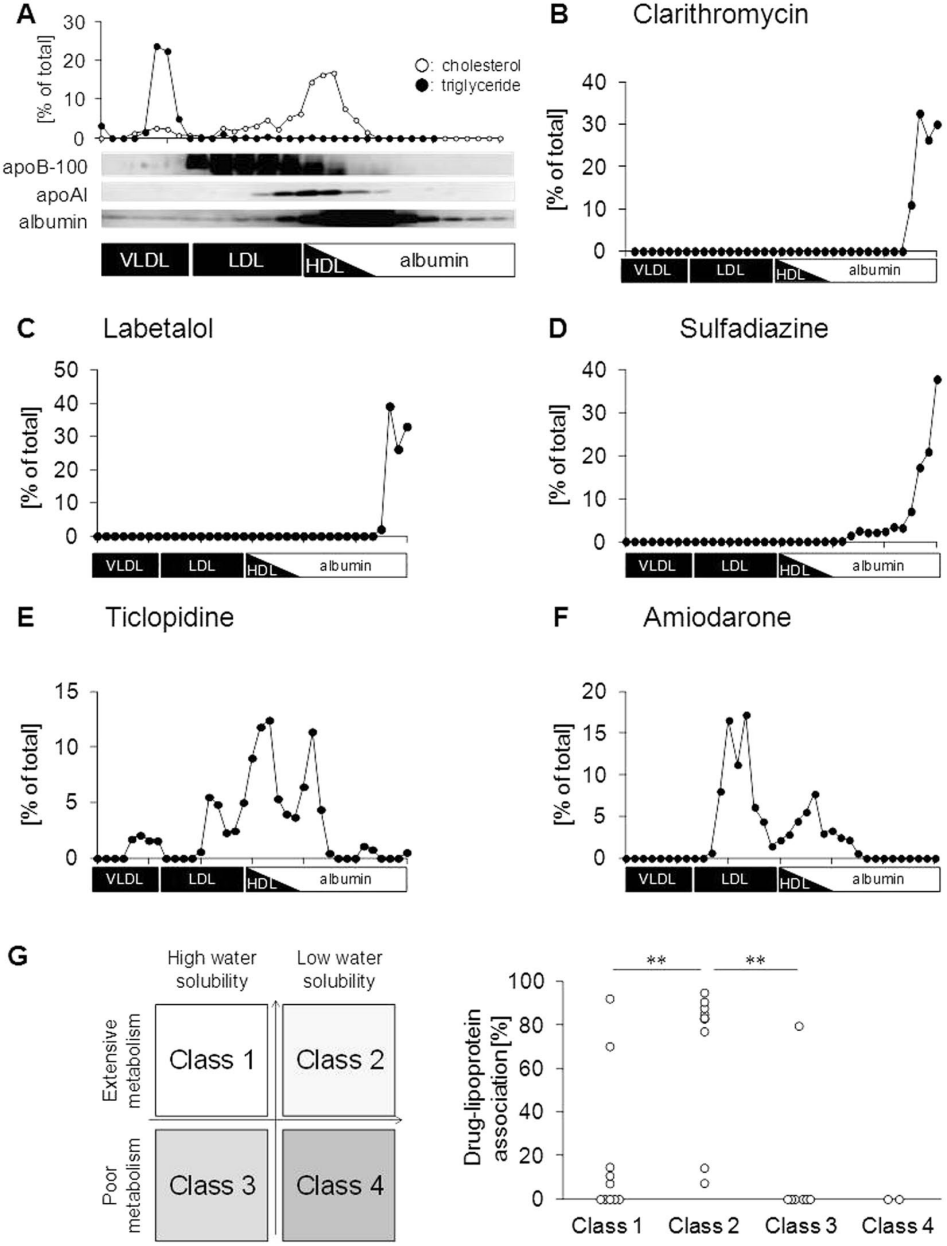
Association of various drugs with lipoproteins. (**A**) Serum lipoprotein fractions from wild-type mice obtained 2 h after oral drug administration were identified according to the concentrations of cholesterol and triglycerides, and the presence of apoB-100, apoAI, and albumin. The total amount of cholesterol and triglycerides in the fractions was considered to be 100%. (**B–F**) *In vivo* analysis of drug association with lipoproteins. (**B**) Clarithromycin; **(C**) labetalol; (**D**) sulfadiazine; (**E**) ticlopidine; and (**F**) amiodarone. (**G**) The left panel shows the BDDCS classification of drugs into four types. The right panel shows the extent of drug-lipoprotein association according to BDDCS. **p < 0.01.

To determine whether drugs associate with lipoproteins *in vivo*, 42 randomly selected drugs were orally administered to mice, and the lipoprotein-associated drug content was determined by quantifying drug concentrations in serum lipoprotein fractions collected by size-exclusion chromatography. Each lipoprotein fraction was identified according to the concentrations of cholesterol and triglycerides, and the presence of apolipoprotein B-100 (apoB-100, a marker of VLDL and LDL), apolipoprotein A-I (apoAI, a marker of HDL), and albumin (Fig. 1A). Drug concentrations in each fraction were quantified using liquid chromatography-tandem mass spectrometry (LC/MS/MS). Consistent with previous reports demonstrating that albumin is a major drug-binding protein in blood^9, 10^, many drugs such as clarithromycin, labetalol, and sulfadiazine were detected in albumin fractions (Fig. 1B–D). However, more than 20% of drugs (10 of 42 drugs), including ticlopidine and amiodarone, were detected mainly in lipoprotein fractions (Fig. 1E and F). We then classified these drugs based on the Biopharmaceutics Drug Disposition Classification System (BDDCS), which classifies drugs into four groups according to their water solubility and extent of drug metabolism by metabolic enzymes such as cytochrome P-450^11^ (Fig. 1G and Supplementary Table S1). Notably, most lipoprotein-associated drugs are categorized as class 2 drugs (7 of 10 lipoprotein-associated drugs), which exhibit low water solubility and extensive metabolism. In addition, we analyzed the extent of drug-lipoprotein association (DLA) by calculating the amount of each drug in lipoprotein fractions for drugs exhibiting more than 90% recovery (with free fraction) after size-exclusion chromatography. The results showed that DLA was significantly higher for class 2 drugs than for the other classes of drugs (Fig. 1G and Supplementary Fig. S2). These findings suggest that lipophilic drugs with extensive hepatic metabolism have a strong tendency to associate with lipoproteins.

### Effect of lipoprotein metabolism modification on the behavior of lipoprotein-associated drugs

**Figure 2:**
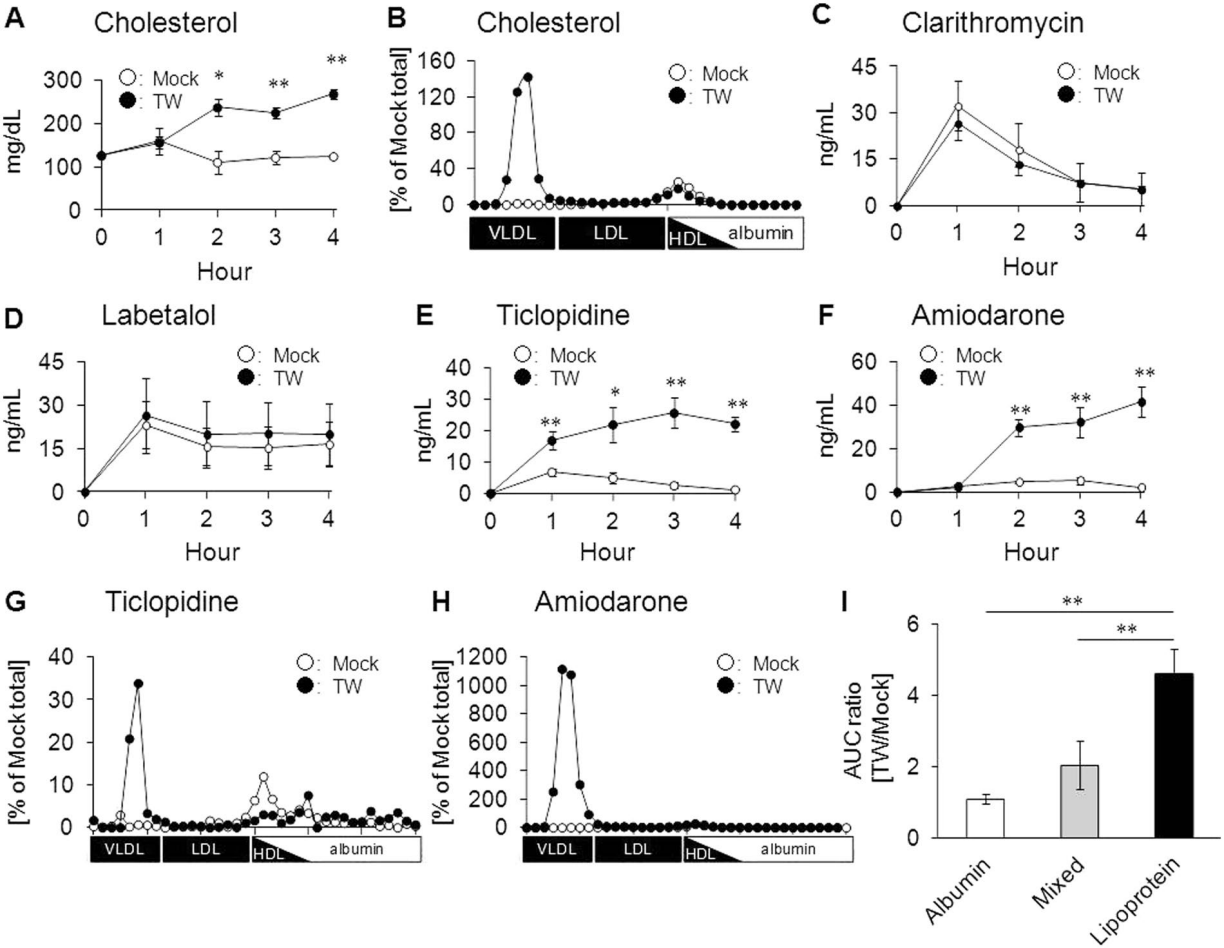
Effect of lipoprotein metabolism modification on the behavior of lipoprotein-associated drugs. **(A**) Serum cholesterol concentrations after intravenous administration of saline (Mock) or Triton WR-1339 (TW), which inhibits the conversion of VLDL to LDL. **(B)** Association of cholesterol with lipoproteins at 4 h after TW treatment. **(C–F**) Serum drug concentrations after TW treatment. (**C**) Clarithromycin; (**D**) labetalol; (**E**) ticlopidine; and (**F**) amiodarone. (**G–H**) Analysis of the association of **(G**) ticlopidine and **(H**) amiodarone with lipoproteins at 4 h after TW treatment. **(I)** AUC ratios for albumin-bound drugs (albumin), lipoprotein-associated drugs (lipoprotein), and mixed-type drugs (mixed). Bars represent the mean ± s.e.m. *p < 0.05, **p < 0.01.

Among lipoproteins, LDL is primarily responsible for delivering cholesterol to peripheral tissues. LDL is converted from VLDL by lipoprotein lipase (LPL), and is then taken up by various tissues via LDLR-mediated endocytosis^12^. Since lipoprotein-associated drugs were detected in VLDL/LDL fractions (Fig. 1E and F), we examined whether modulation of VLDL/LDL metabolism could affect the behavior of lipoprotein-associated drugs. For this purpose, we used Triton WR-1339 (TW), an LPL inhibitor, which causes accumulation of VLDL in the bloodstream *in vivo* ^13^. Intravenous injection of TW to mice increased the serum concentrations of cholesterol and triglycerides (Fig. 2A and Supplementary Fig. S3A) because of the accumulation of these lipids in VLDL fractions (Fig. 2B and Supplementary Fig. S3B). Under this condition, we analyzed the serum concentrations of three types of drugs following oral administration: an albumin-bound type with a DLA of <10%, lipoprotein-associated type with a DLA of >50%, and a mixed type with a DLA of 10–50%. The results showed that TW treatment had no significant effect on the serum concentrations of albumin-bound drugs such as clarithromycin and labetalol (Fig. 2C and D). However, the serum concentrations of lipoprotein-associated drugs such as ticlopidine and amiodarone were significantly higher in TW-administrated mice than in Mock-treated mice (Fig. 2E and F), which could be accounted for by drug accumulation in VLDL fractions (Fig. 2G and H). Calculation of the area under the serum concentration-time curve (AUC) for each type of drug and determination of the AUC ratio of TW-treated mice to Mock-treated mice showed that drugs with a higher DLA had a higher AUC ratio (Fig. 2I). Indeed, the AUC ratios of lipoprotein-associated drugs were significantly higher than those of albumin-bound drugs. These findings indicate that lipoprotein-associated drugs are more sensitive to changes in VLDL/LDL metabolism than albumin-bound drugs. Taken together, our results indicated that VLDL/LDL influences the behavior of lipoprotein-associated drugs. Since TW treatment markedly increased the serum concentrations of P2Y12 receptor antagonists, including ticlopidine, ticagrelor, and clopidogrel (Supplementary Fig. S3C), these drugs were used in further experiments.

### Effect of LDLR overexpression on LDL-mediated drug transport *in vitro*

**Figure 3:**
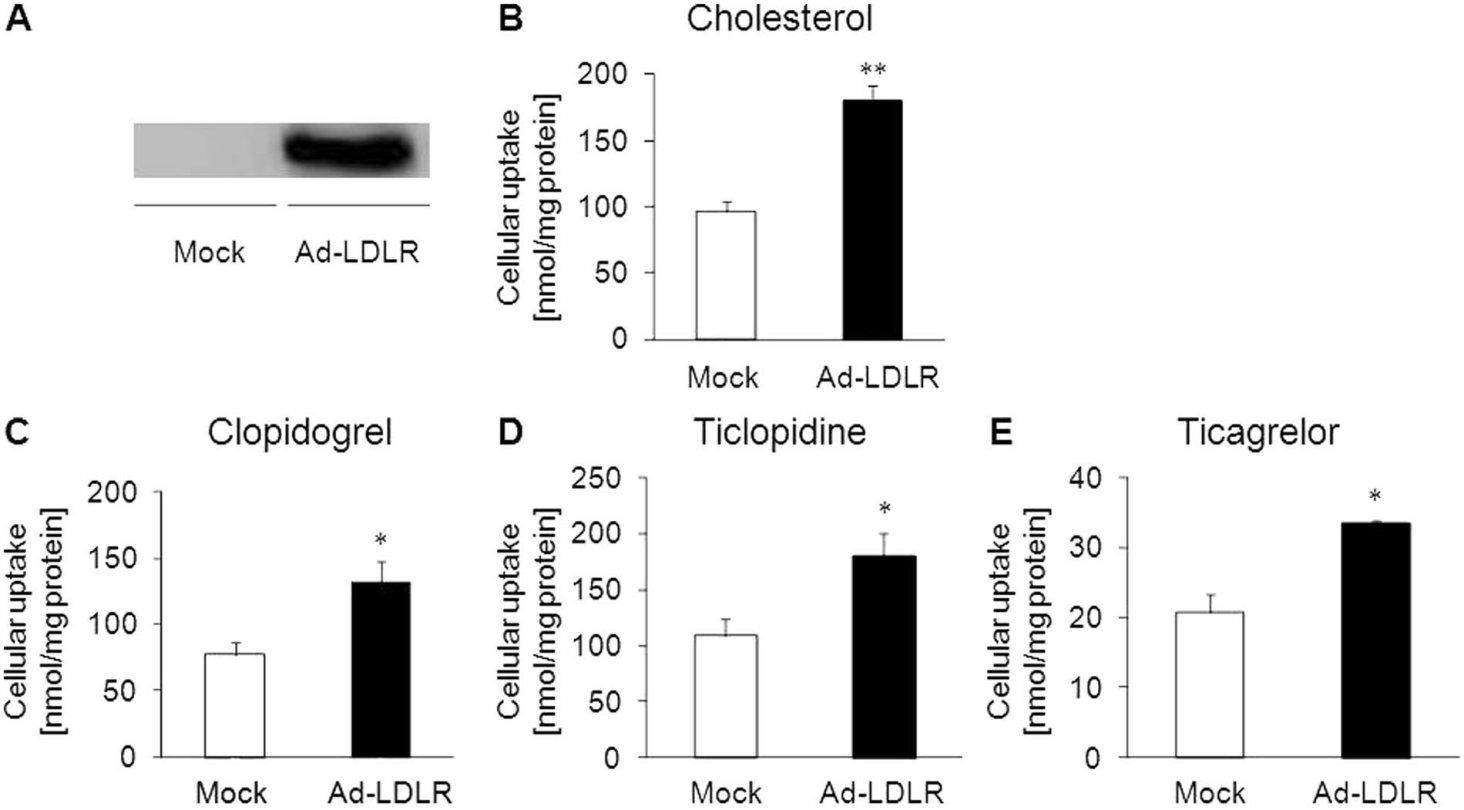
Effect of LDLR overexpression on LDL-mediated drug transport *in vitro*. (**A**) Hepa1-6 cells were infected with green fluorescent protein (GFP)-tagged mouse LDLR-expressing adenovirus (Ad-LDLR). Two days after infection, LDLR-GFP overexpression was confirmed by western blotting. GFP-expressing adenovirus (Mock) was used as a control. **(B)** The uptake of LDL solution containing [^3^H]cholesterol in FBS-free transport buffer was measured in Mock and Ad-LDLR cells. **(C–E)** *In vitro* uptake experiments for LDL-associated **(C**) clopidogrel, (**D**) ticlopidine, and (**E**) ticagrelor in Mock and Ad-LDLR cells. Bars represent the mean ± s.e.m. *p < 0.05, **p < 0.01.

Next, to clarify the role of LDLR in mediating VLDL/LDL-associated drug transport, we performed drug uptake experiments using LDLR-overexpressing Hepa1-6 cells, a mouse liver hepatoma cell line (Fig. 3A). We first confirmed that the uptake of [^3^H]cholesterol in LDL and that of fluorescent-labeled LDL were higher in LDLR-overexpressing cells than in Mock-treated cells (Fig. 3B and Supplementary Fig. S4), which indicated the utility of this experimental system to assess LDLR-mediated uptake of LDL particles. We then performed drug uptake experiments and revealed that LDLR overexpression significantly increased the uptake of LDL-associated P2Y12 receptor antagonists (Fig. 3C–E).

### Effect of LDLR knockdown on LDL-mediated drug transport *in vitro*

**Figure 4:**
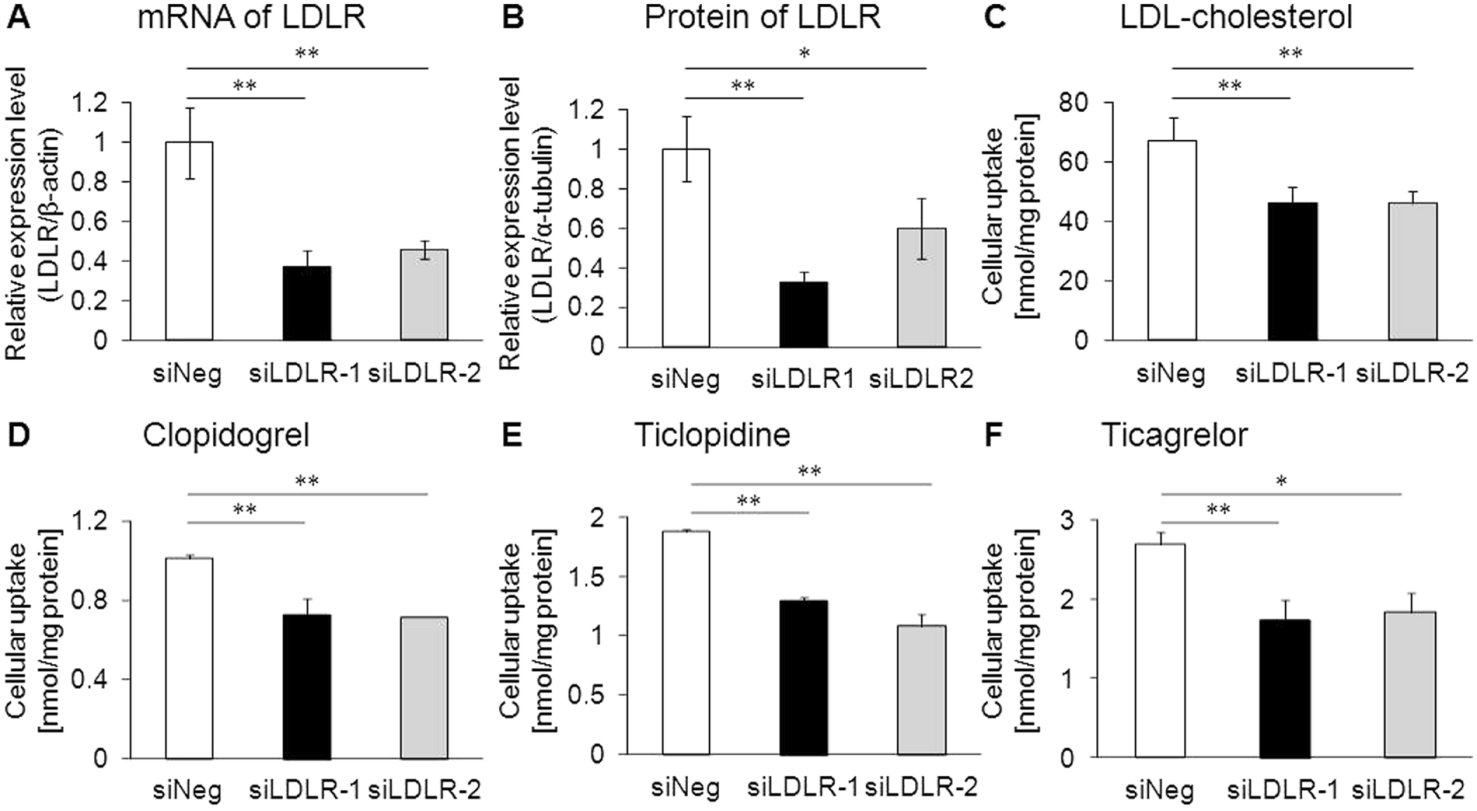
Effect of LDLR knockdown on LDL-mediated drug transport *in vitro*. **(A)** Messenger RNA levels and **(B)** protein levels of LDLR in control (siNeg) and LDLR-knockdown (siLDLR-1 and siLDLR-2) cells as determined by quantitative real-time PCR and western blotting, respectively. **(C)** The uptake of reconstituted LDL containing [^3^H]cholesterol in FBS-free transport buffer was measured in siNeg, siLDLR-1, and siLDLR-2 cells. **(D–F)** *In vitro* uptake experiments of **(D**) clopidogrel, (**E**) ticlopidine, and (**F**) ticagrelor in reconstituted LDL in siNeg, siLDLR-1, and siLDLR-2 cells. Bars represent the mean ± s.e.m. *p < 0.05, **p < 0.01.

To obtain further evidence of VLDL/LDL-mediated drug transport, drug uptake experiments were performed using LDLR-knockdown Huh-7 cells, a human-derived hepatic cell line. Two different LDLR-knockdown cell lines were generated by transfecting Huh-7 cells with siRNAs against different regions of the human LDLR mRNA (siLDLR-1 and siLDLR-2). The mRNA (Fig. 4A) and protein (Fig. 4B and Supplementary Fig. S5A and B) levels of LDLR were significantly decreased in both cell lines. *In vitro* drug uptake experiments using these cell lines showed that, similar to the uptake of [^3^H]cholesterol in LDL (Fig. 4C), the uptake of LDL-associated drugs was significantly lower in the two LDLR-knockdown cell lines (siLDLR-1 and siLDLR-2) than in control cells (siNeg) (Fig. 4D–F). These *in vitro* results suggest that LDL-associated drugs can be taken up by LDLR-mediated endocytosis.

### Clarification of VLDL/LDL-mediated drug transport *in vivo*

**Figure 5:**
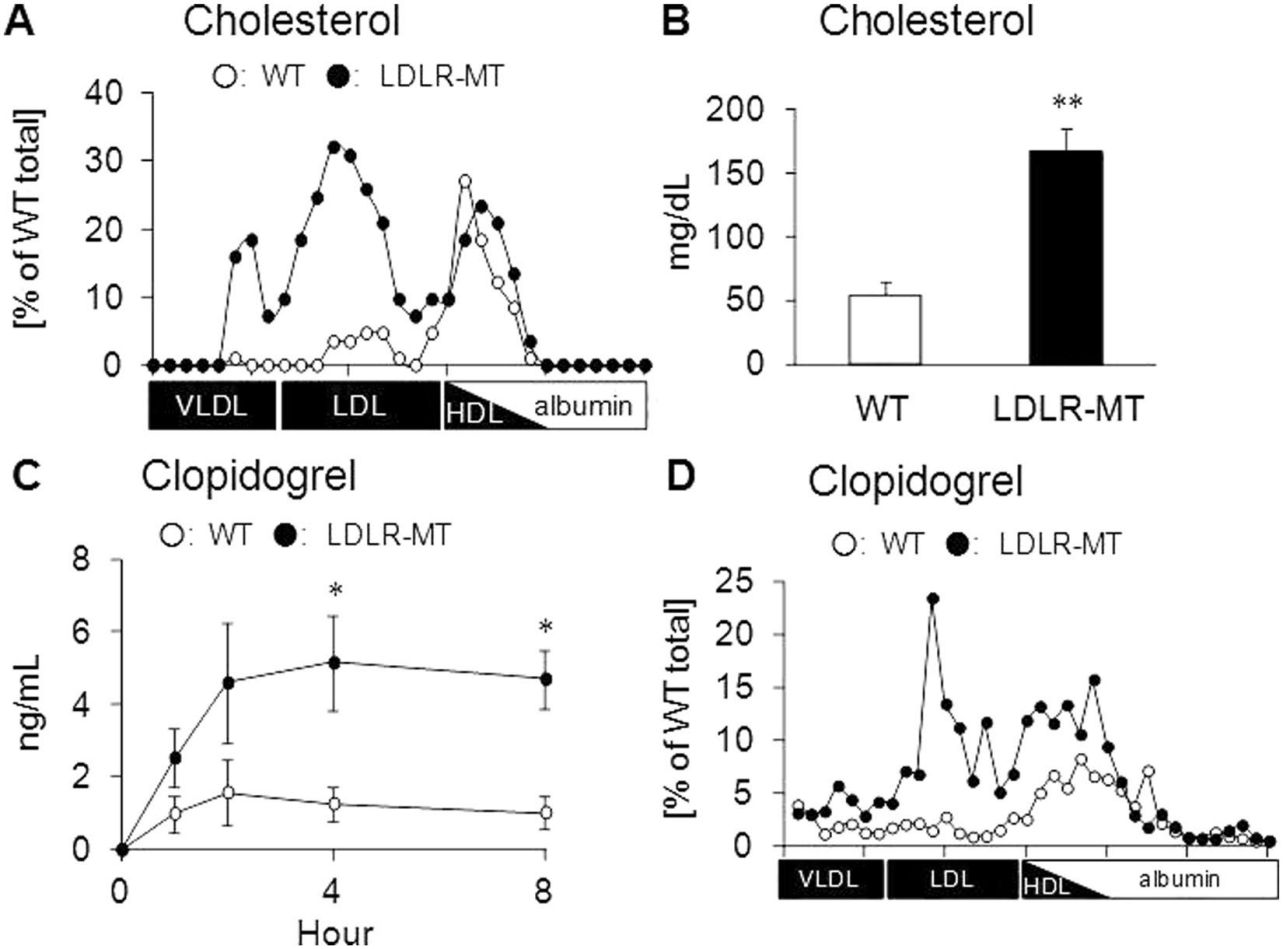
Clarification of VLDL/LDL-mediated drug transport *in vivo*. **(A)** Comparison of the association of cholesterol with lipoproteins between wild-type (WT) and LDLR-mutant (LDLR-MT) mice. **(B)** Serum cholesterol concentrations in WT and LDLR-MT mice. **(C)** Serum clopidogrel concentrations in WT and LDLR-MT mice. **(D)** Clopidogrel concentrations in lipoprotein fractions at 4 h after clopidogrel administration. Bars represent the mean ± s.e.m. *p < 0.05. **p < 0.01.

To determine whether LDLR affects the behavior of lipoprotein-associated drugs *in vivo*, the serum concentrations of lipoprotein-associated drugs were analyzed in LDLR-MT mice in which the LDLR function is defective. Similar to LDLR knockout mice^14^, LDLR-MT mice accumulated cholesterol and triglycerides in VLDL/LDL fractions (Fig. 5A and Supplementary Fig. S6A), which resulted in higher serum concentrations of both lipids in LDLR-MT mice than in wild-type (WT) mice (Fig. 5B and Supplementary Fig. S6B). In addition to these lipids, the serum concentrations of clopidogrel were significantly higher in LDLR-MT mice than in WT mice after oral administration (Fig. 5C), which was caused by the accumulation of clopidogrel in VLDL/LDL fractions in LDLR-MT mice (Fig. 5D). By contrast, drugs that were detected mainly in albumin fractions, such as tolbutamide, showed no significant differences in the serum concentration (Supplementary Fig. S6C) or drug association with serum fractions between LDLR-MT and WT mice (Supplementary Fig. S6D). These results strongly suggest that LDLR regulates the behavior of lipoprotein-associated drugs but not that of albumin-bound drugs.

### Effect of LDLR overexpression on VLDL/LDL-mediated drug transport *in vivo*

**Figure 6:**
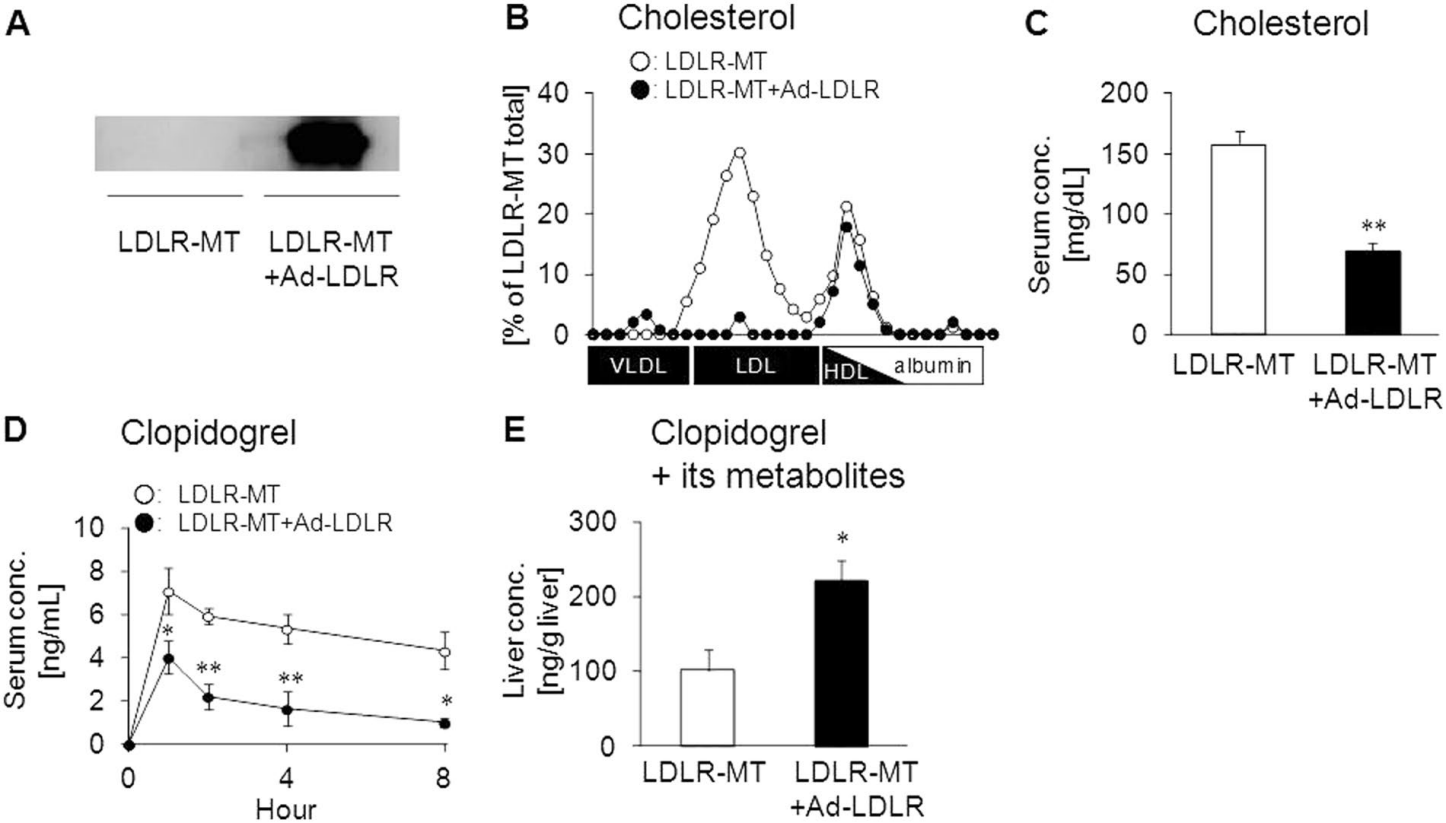
Effect of LDLR overexpression on VLDL/LDL-mediated drug transport *in vivo*. **(A**) LDLR-mutant (LDLR-MT) mice were infected with LDLR-expressing adenovirus to generate LDLR-rescued mice (LDLR-MT+Ad-LDLR). GFP-expressing adenovirus was used as a control. Three days after the infection, overexpression of LDLR was confirmed by western blotting. **(B)** Association of cholesterol with lipoproteins in LDLR-MT and LDLR-MT+Ad-LDLR mice. **(C)** Effect of adenovirus-mediated LDLR-overexpression (Ad-LDLR) on serum cholesterol concentration in LDLR-MT mice. **(D)** Serum clopidogrel concentration in LDLR-MT and LDLR-MT+Ad-LDLR mice. **(E)** Effect of LDLR rescue on the hepatic concentration of clopidogrel and its metabolites at 4 h after clopidogrel administration. Bars represent the mean ± s.e.m. *p < 0.05, **p < 0.01.

To further confirm that LDLR plays a key role in lipoprotein-associated drug uptake *in vivo*, we performed a LDLR rescue experiment using an adenovirus-mediated gene transfer system to overexpress LDLR in the liver of LDLR-MT mice (Fig. 6A). In addition to reduced lipid accumulation in VLDL/LDL fractions (Fig. 6B and Supplementary Fig. S7A), the serum concentrations of cholesterol (Fig. 6C) and triglycerides (Supplementary Fig. S7B) were lower in LDLR-rescued mice than in control mice, indicating that the transduced LDLR was functional in the liver of LDLR-MT mice. Analysis of serum and liver clopidogrel concentrations in LDLR-rescued mice following oral drug administration showed that serum clopidogrel was significantly reduced by LDLR rescue (Fig. 6D), whereas the total concentrations of clopidogrel and its metabolites in the liver were increased (Fig. 6E). These results indicated that clopidogrel accumulated in VLDL/LDL fractions in LDLR-MT mice can be taken up by the liver when functional LDLR is expressed, suggesting that LDLR plays a key role in the uptake of VLDL/LDL-associated drugs *in vivo*.

### Effect of lipoprotein apheresis treatment on VLDL/LDL-associated drug behavior in patients with familial hypercholesterolemia

**Figure 7:**
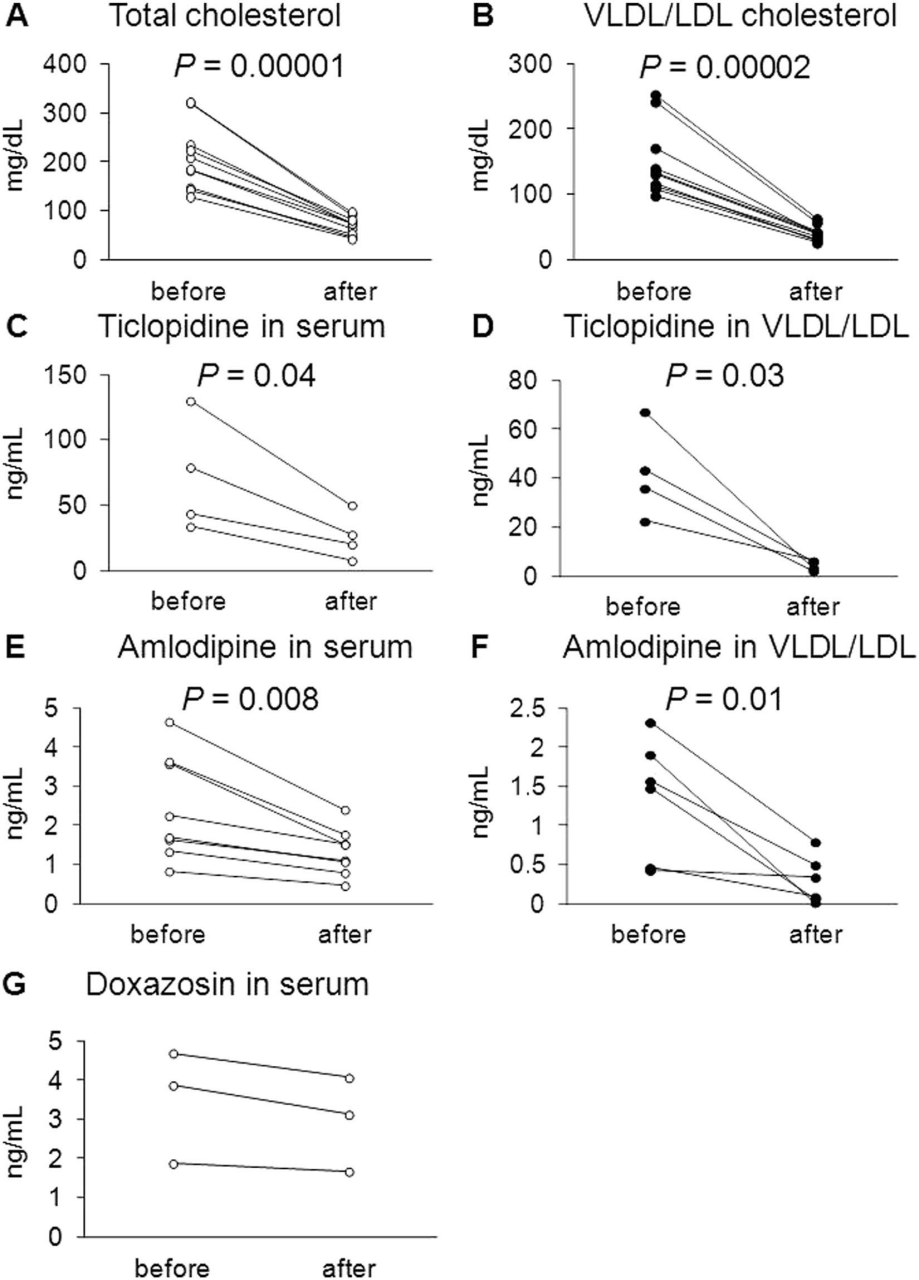
Effect of lipoprotein apheresis treatment on VLDL/LDL-associated drug behavior in patients with familial hypercholesterolemia. (**A**) Total cholesterol concentration in serum before and after lipoprotein apheresis treatment. (**B**) VLDL/LDL cholesterol concentration in serum before and after lipoprotein apheresis treatment. (**C**,**E**, and **G**) Drug concentrations in serum before and after lipoprotein apheresis treatment. (**C**) Ticlopidine; (**E**) amlodipine; and (**G**) doxazosin. (**D**,**F**) VLDL/LDL-associated drug concentrations before and after lipoprotein apheresis. (**D**) Ticlopidine and (**F**) amlodipine. (**A–F**) *P* values were determined using paired *t* test.

Finally, to examine whether VLDL/LDL-mediated drug transport can influence drug behavior in humans, we performed a clinical study analyzing serum drug concentrations before and after lipoprotein apheresis treatment in patients with familial hypercholesterolemia (FH). FH is characterized by the accumulation of VLDL/LDL particles in the blood because of genetic dysfunction of LDLR or LDLR-related genes^15^. Therefore, lipoprotein apheresis treatment is important to prevent the onset of atherosclerosis and cardiovascular disease in patients with FH^16–19^. Lipoprotein apheresis treatment can selectively remove VLDL/LDL cholesterol from blood through the electrostatic interaction of dextran sulfate in the lipoprotein apheresis column with apoB on (V)LDL particles^20^. Therefore, if VLDL/LDL-mediated drug transport occurs in humans, the serum concentrations of VLDL/LDL-associated drugs would be decreased after lipoprotein apheresis treatment. To test this possibility, we recruited FH patients who were treated with certain drugs including lipoprotein-associated drugs. Lipoprotein apheresis treatment decreased cholesterol levels in the serum and VLDL/LDL fractions by 67% and 73%, respectively, in the recruited patients (Fig. 7A and B). Quantification of drug concentrations in serum and VLDL/LDL fractions before and after lipoprotein apheresis treatment showed that, similar to the decrease in cholesterol concentration, serum ticlopidine concentration decreased by 64% in response to lipoprotein apheresis treatment (Fig. 7C). A marked decrease in the concentration of ticlopidine by 87% was observed in VLDL/LDL fractions in response to lipoprotein apheresis treatment (Fig. 7D), which accounted for the decrease in serum ticlopidine concentration. Similar to ticlopidine, amlodipine decreased by 46% (Fig. 7E) and 71% (Fig. 7F) in the serum and VLDL/LDL fractions, respectively, with lipoprotein apheresis treatment. On the other hand, lipoprotein apheresis hardly affected the serum concentration of doxazosin (Fig. 7G), which was consistent with the observation that doxazosin was not detected in VLDL/LDL fractions. Considering that the half-life of doxazosin (22 h) is shorter than that of ticlopidine (4–5 days) and amlodipine (30–50 h), these results may indicate that VLDL/LDL can play an important role as a carrier for lipoprotein-associated drugs as well as for lipids in humans.

## Discussion

In the present study, we demonstrated that LDL functions as a carrier for drugs as well as lipids such as cholesterol and triglycerides (Figs 1 and 2). In addition, we also showed that VLDL/LDL-associated drugs are taken up by tissues in an LDLR-dependent manner (Figs 3–6). These findings indicate that, in addition to free drug concentrations in serum, the association of drug with lipoproteins should also be considered to understand the behavior of drugs *in vivo*. Humans have a higher serum concentration of LDL than mice because cholesteryl ester transfer protein, which is involved in the transfer of cholesteryl ester from HDL to LDL in blood, is functional in humans but not in mice^21^. Considering that human LDLR can function as a receptor for drug-laden LDL (Fig. 4) and that lipoproteins can function as drug carriers in humans (Fig. 7), it is possible that VLDL/LDL-mediated drug transport has a stronger impact on the behavior of lipoprotein-associated drugs in humans than that expected from the results of *in vivo* experiments in mice.

Consistent with previous reports that lipoproteins can deliver certain lipophilic compounds such as fat-soluble vitamins and endocrine-disrupting substances^6–8^, we demonstrated that class 2 drugs exhibiting low water solubility have a strong tendency to associate with lipoproteins (Fig. 1G and Supplementary Fig. S2). However, some class 2 drugs, such as glimepiride, were detected mainly in the albumin-bound fractions (Supplementary Fig. S2B). In addition, certain class 1 drugs showing high water solubility, such as ticlopidine and amlodipine, can associate with VLDL/LDL particles in mice and humans (Figs 1 and 7). These observations indicate that, although class 2 drugs have a tendency to associate with VLDL/LDL particles, the extent of DLA does not always depend on drug lipophilicity, and other unknown factors may regulate the drug association with lipoproteins. A detailed characterization of lipoprotein-associated drugs is necessary to clarify these factors in the future.

We showed that modulation of lipoprotein behavior in the body can alter the behavior of lipoprotein-associated drugs (Figs 2, 5 and 6). This indicates that drug dose adjustments according to lipid status in each patient would be useful for effective personalized medicine. In particular, the fact that some lipid-lowering drugs can affect not only the behavior of lipoproteins but also that of lipoprotein-associated drugs is important. Inhibitors of proprotein convertase subtilisin/kexin type 9 (PCSK9) have recently been developed intensively and are being introduced clinically as a new class of lipid-lowering drugs for the treatment of hyperlipidemia^22^. PCSK9 inhibitors prevent the degradation of LDLR, which results in increased (V)LDL uptake from blood mainly by the liver and decreased plasma LDL- cholesterol levels^23, 24^. Our finding that LDLR can function as a receptor for drug-laden LDL (Figs 3–6) suggests that the increase in the LDLR-mediated (V)LDL uptake caused by PCSK9 inhibitors can also alter the behavior of lipoprotein-associated drugs in the body. Clinical studies aimed at elucidating the behavior of lipoprotein-associated drugs in patients treated with PCSK9 inhibitors would be important to reveal novel drug-drug interactions mediated by alterations in lipoprotein clearance.

Lipoprotein apheresis treatment is vital to FH patients because the presence of abnormally high LDL cholesterol leads to the formation of atherosclerotic plaques and increases the risk of cardiovascular diseases^16–19^. Lipoprotein apheresis treatment is currently believed to selectively eliminate VLDL/LDL cholesterol^20^. However, we showed that lipoprotein apheresis treatment unintentionally removes lipoprotein-associated drugs from serum VLDL/LDL fractions (Fig. 7). Since FH patients take various medicines including lipoprotein-associated anti-platelet drugs to prevent cardiovascular events, our findings are important to improve the efficacy of drug therapy for these patients. The administration of medicine after lipoprotein apheresis treatment rather than before would be helpful to maintain serum drug concentrations within the therapeutic range, which could benefit drug therapy.

Although our aim was to clarify VLDL/LDL-mediated drug transport as an important factor affecting drug transport and metabolism after oral administration, HDL should also be considered as a drug carrier. Indeed, clopidogrel and ticlopidine were detected in HDL fractions after oral administration of these drugs (Figs 1E and 5D). In addition, clozapine was reported to be present in the HDL fraction in humans^25^. These results confirm the association between HDL and drugs. Since the behavior of HDL is regulated by several membrane proteins, such as scavenger receptor type B class I^26^, ATP-binding cassette transporter A1 (ABCA1)^27^, and ABCG1^28^, pharmacokinetic and/or pharmacodynamic analyses in mice with (genetically) modified expression of these proteins will be useful to clarify the importance of HDL-mediated drug transport *in vivo*.

Unlike the unbound free form, lipoprotein-associated drugs cannot be discriminated from protein-bound forms by conventional separation methods such as dialysis and ultrafiltration. Therefore, lipoprotein-associated and protein-bound forms are usually considered together when predicting drug behaviors. However, based on our results, the lipoprotein-associated form should be newly defined as a distinct form of drugs, as its behaviors differs from that of the protein-bound form *in vivo* (Supplementary Fig. S1). Precise evaluation of free, protein-bound, and lipoprotein-associated drug concentrations may enable better predictions of drug behaviors.

In conclusion, we identified lipoproteins as drug carriers, providing new insight into the regulation of drug behavior. The results of our clinical study demonstrating that lipoprotein apheresis treatment can affect the behavior of lipoprotein-associated drugs support the generality and importance of our findings in humans. According to the BDDCS drug classification system (Fig. 1), approximately 70% of new drugs in clinical trials, in addition to 30% of already-available drugs, are estimated to be class 2 drugs^11^. This observation, together with our findings that class 2 drugs are more likely to associate with lipoproteins than other classes of drugs (Fig. 1), suggests that a novel concept of lipoprotein-mediated drug transport will be of increasing importance for effective and safe pharmacotherapy in the near future.

## Materials and Methods

### Materials

Clopidogrel was purchased from LKT Laboratories (St. Paul, MN). Ticlopidine and olive oil were purchased from Wako (Osaka, Japan). Ticagrelor was purchased from Sequoia Research Products Ltd (Oxford, UK). All other chemicals used were commercially available and of reagent grade.

### Animals

Male ddY mice were purchased from Japan SLC, Inc. LDLR-MT (C146R) mice strain (RBRC-GSC0247) were provided by RIKEN BRC through the National Bio-Resource Project of MEXT (Tokyo, Japan). All animals were housed in temperature- and humidity-controlled animal cages with a 12-hour dark-light cycle and with free access to water and standard animal chow (FR-1, Funabashi Farm, Funabashi, Japan).

### Ethics statement

All animal experiments were conducted using protocols approved by the Institutional Animal Care Committee of the University of Tokyo. All animals received humane care according to the criteria outlines in the Guide for the Care and Use of Laboratory Animals prepared by the National Academy of Sciences and published by the National Institutes of Health.

### Fractionation of lipoproteins and albumin from serum

Mouse serum was fractionated by size-exclusion chromatography using a Pharmacia Smart System^®^ fast protein liquid chromatography (FPLC) system equipped with a Superose 6 column (GE Healthcare, Milwaukee, WI), according to previously described methods^29^. Elution was performed in phosphate buffered saline (PBS) containing 1 mM EDTA and 3 mM sodium azide as a running buffer. After loading 300 μL of serum, the system was run with a constant flow of 200 μL/min with 500 μL of fractions. Fractions 12–36 containing serum lipoproteins and fractions 37–48 containing serum albumin were used for further analyses.

### Quantification of cholesterol and triglycerides concentrations

The concentrations of cholesterol and triglycerides were quantified using a Cholesterol E-test WAKO (Wako) and a Triglyceride E-test WAKO (Wako), respectively.

### Immunoblot analysis

ApoB-100, apoAI, albumin, α-tubulin, and GFP-tagged mouse LDLR and human LDLR expression was determined by western blotting. For separation, 7% SDS-polyacrylamide gels were used for apoB-100, albumin, α-tubulin, and LDLR, whereas 10% SDS-polyacrylamide gels were used for apoAI. Molecular weights were determined by reference to a prestained protein ladder (Gene DireX, Taipei, Taiwan). Primary antibodies used for experiments were rabbit anti-apoB-100 (Acris Antibodies GmbH, Herford, Germany; diluted 500-fold), rabbit anti-apoAI (Santa Cruz Biotechnology, Inc., CA; diluted 200-fold), goat anti-albumin (Santa Cruz Biotechnology, Inc.; diluted 500-fold), rabbit anti-α-tubulin (Abcam, Cambridge, MA; diluted 1,000-fold) and rabbit anti-GFP (Life Technologies, Gaithersburg, MD; diluted 2,000-fold), and rabbit anti-LDLR (Novus, Littleton, CO; diluted 100-fold). For detection, membranes were incubated with horseradish peroxidase-labeled secondary antibody (anti-rabbit IgG (GE Healthcare) or anti-goat IgG (Santa Cruz Biotechnology, Inc.)) diluted 5,000-fold in TBS-T (TBS-Tween20) containing 0.1% bovine serum albumin (BSA) for 1 h at room temperature. Proteins were visualized using an ECL Prime Western Blotting Detection System (GE Healthcare) with a luminescent image analyzer (Bio-Rad Laboratories, Tokyo, Japan). Full length figures of membranes are shown in Supplementary Figs S5 and S8.

### Sample preparation for drug quantification

Serum and liver samples were mixed with an internal standard solution (100 ng/mL pioglitazone in 30% ethanol), followed by deproteinization with four volumes of methanol and four volumes of acetonitrile. After centrifugation at 15,000 rpm for 5 min, drug concentrations in the supernatants were analyzed by LC/MS/MS. For analysis of fractionated serum samples, deproteinized supernatants were concentrated using a Speed-Vac concentrator (Kubota, Tokyo, Japan). Drug concentrations in the concentrated solutions were then quantified by LC/MS/MS.

### Quantification of drugs by LC/MS/MS

LC/MS/MS multiple reaction monitoring analyses were conducted on a XEVO™ Tandem Quadrupole Mass Spectrometer coupled to an ACQUITY Ultra Performance LC (UPLC) System with an integral autoinjector (Waters, Milford, MA). The XEVO spectrometer was run in electrospray ionization-MS/MS multiple reaction monitoring mode at a source temperature of 120 °C and a desolvation temperature of 350 °C. Sample temperature was kept at 4 °C, and column temperature was kept at 50 °C. The mobile phases were 0.1% formic acid solution (solvent A) and liquid chromatography-grade acetonitrile (Sigma Aldrich, Inc., St Louis, MO) (solvent B). The UPLC conditions and mass spectrometer conditions are shown in Supplementary Table S2. Data analyses were performed using MassLynxNT software (version 4.1) (Waters).

Drug association with lipoproteins and albumin was calculated by dividing the drug content in the lipoprotein or albumin fraction by the injected serum drug content. The free fraction was obtained from the DrugBank database^30^. In cases in which the sum of the drug association with lipoprotein, albumin, and free fractions was more or less than 100%, the sum of each association rate was adjusted to 100%.

### Triton WR-1339 treatment

Male ddY mice at 6–8 weeks of age were orally administered with 5 mg/kg of each drug in olive oil after intravenous injection of saline or 500 mg/kg TW (Sigma Aldrich, Inc.). Four hours after oral drug administration, mouse serum was collected and fractionated by FPLC to analyze the association of each drug with lipoproteins as described above.

### Construction of expression vectors and recombinant adenoviruses

Mouse LDLR complementary DNA (Accession Number: NM010700.3) was inserted into a pEGFP-N1 plasmid vector (Life Technologies) to express GFP-tagged mouse LDLR (LDLR-GFP). Recombinant adenoviruses expressing LDLR-GFP were prepared using the ViraPower™ Adenoviral Expression system (Life Technologies) according to the manufacturer’s instructions and purified by cesium chloride gradient centrifugation. GFP-expressing adenoviruses were purified with the same method. Viral titers (plaque-forming units per milliliter) were determined using an Adeno-X rapid titer kit (Takara Bio, Tokyo, Japan), and multiplicity of infection (MOI) was determined by normalizing the viral titer to the cell count in each experiment.

### Production of reconstituted LDL

Reconstituted LDL solutions were prepared as described previously^31–33^. Briefly, LDL was separated from fetal bovine serum (FBS; Biowest, Nuaillé, France) by KBr density gradient ultracentrifugation. After quantifying the protein concentration of the separated LDL solution, 22.8 mg of starch was added to 1.9 mg of protein in the LDL solution. The solvent was then evaporated under reduced pressure. After evaporation, the lipid component in LDL was extracted twice with heptane. The residue was supplemented with 5 mg of cholesteryl linoleate and proper amounts of drugs or [^3^H]cholesterol in chloroform, followed by incubation for 1 h on ice. The solvent was then evaporated under nitrogen gas, and 1 mL of transport buffer (118 mM NaCl, 23.8 mM NaHCO_3_, 4.4 mM KCl, 0.96 mM KH_2_PO_4_, 1.2 mM MgSO_4_, 12.5 mM HEPES, 5 mM glucose, and 1.5 mM CaCl_2_ at pH 7.4) was added to the residue, followed by incubation at 4 °C for 12 h. Finally, the starch was removed by centrifugation, and the supernatant was used for experiments.

### LDLR-mediated uptake assay

Mouse hepatoma Hepa1-6 cells were cultured in Dulbecco’s Modified Eagle’s Medium (DMEM) (Nacalai Tesque, Tokyo, Japan) containing 10% FBS and 1% penicillin streptomycin (Nacalai Tesque) at 37 °C with 5% CO_2_.

Hepa1-6 cells were seeded on 12-well plates at a density of 2.0 × 10^5^ cells/well. Twenty-four hours after seeding, GFP-expressing adenovirus or LDLR-GFP-expressing adenovirus was infected at 100 MOI. Forty-eight hours after adenovirus infection, uptake assays for cholesterol and various drugs were performed. Cells were incubated with reconstituted LDL containing 1 μM of each drug or 670 pM [^3^H]cholesterol at 37 °C. After incubation for 2 h, cells were washed twice with transport buffer. Cells were solubilized in 500 μL of 0.2 N NaOH overnight. Cell lysates were neutralized with 100 μL of 1 N HCl. Radioactivity was then measured using a liquid scintillation counter to quantify the uptake of [^3^H]cholesterol. Quantification of the lysate drug concentration was performed using LC/MS/MS as described above.

### Fluorescent-labeled LDL uptake assay

The fluorescent-labeled LDL uptake assay was performed according to the manual of the LDL Uptake Cell-Based Assay Kit (Cayman, Ann Arbor, MI). Hepa1-6 cells were cultured in DMEM containing 10% FBS and 1% penicillin streptomycin at 37 °C with 5% CO_2_.

Hepa1-6 cells were seeded on 96-well plates at a density of 1.0 × 10^5^ cells/well. Twenty-four hours after seeding, GFP-expressing adenovirus or LDLR-GFP-expressing adenovirus was infected at 100 MOI. Forty-eight hours after adenovirus infection, fluorescent-labeled LDL uptake assays were performed. Cells were incubated with fluorescent-labeled LDL diluted 100-fold in FBS-free DMEM. After incubation for 24 h, cells were washed twice with transport buffer. Cells were solubilized in RIPA buffer (0.1% SDS, 0.5% deoxycholate, 1% NP40, 150 mM NaCl, and 20 mM Tris HCl at pH 7.4). Fluorescence (excitation/emission = 540/570 nm) was measured by Fusion Solo 4 (Vilber-Lourmat, Collegien, France).

### Analysis of LDLR-mutant mice

Mice at 6–10 weeks of age were analyzed in each experiment. For the LDLR rescue experiment, 3.0 × 10^9^ (ifu/mouse) of LDLR-GFP-expressing adenovirus was injected intravenously into LDLR-MT mice. As a control (Mock) virus, GFP-expressing adenovirus was administered in the same manner. Three days after viral infection, mice were used for pharmacokinetic analysis. Serum samples were collected at 0, 1, 2, 4, and 8 h after oral administration of 5 mg/kg of each drug in olive oil. Liver samples were collected after 8 h. Drug concentrations in the serum or lipoprotein fractions were analyzed as described above. The livers of mice were homogenized in saline solution at 0.2 g/mL. Clopidogrel and its major metabolite, 2-oxo clopidogrel (Toronto Research Chemicals, Ontario, Canada), were quantified by LC/MS/MS after sample preparation as described above.

### LDLR knockdown

Huh-7, human hepatocarcinoma cells, were cultured in RPMI 1640 medium (Nacalai Tesque) containing 10% FBS and 1% penicillin streptomycin at 37 °C with 5% CO_2_.

Huh-7 cells were suspended in RPMI 1640 medium to a density of 2.0 × 10^5^ cells/mL. Approximately 30 pmol/well of human LDLR siRNA no. 1 (siLDLR-1) (5′-CCACUUGUAGGAGAUGCAUTT-3′), human LDLR siRNA no. 2 (siLDLR-2) (5′-CAGAGGAAAUGAGAAGAAGCTT-3′), or a control siRNA (siNeg) designed not to interfere with any human genes (Sigma Aldrich, Inc.) was added to each 12-well plate in a volume of 100 μL of DMEM. Three microliters of Lipofectamine 2000 (Life Technologies) was then added to each well containing the diluted siRNAs. After incubation for 20 min at room temperature, 900 μL of the cell dilution was added to each well. After incubating for 3 days, cells were harvested and the mRNA and protein levels of human LDLR were determined by quantitative real-time PCR and immunoblot analysis, respectively. Drug uptake experiments using LDLR-knockdown cells were performed as described above.

### Quantitative real-time PCR

To determine the mRNA levels of human LDLR, cells were transfected with siNeg or siLDLR, and then harvested using RNA iso Plus (Takara Bio). Prepared RNA was reverse-transcribed using ReverTra Ace qPCR RT Master Mix (Toyobo, Osaka, Japan), and quantitative real-time PCR was performed. Primers used were as follows: LDLR sense (5′-TCTGTCGTGTGTGTTGGGAT-3′), LDLR antisense (5′-ACGACAAGATTGGGGAAGTG-3′), β-actin sense (5′-CCGGAAGGAAAACTGACAGC-3′), and β-actin antisense (5′-GTGGTGGTGAAGCTGTAGCC-3′).

### Analysis of drug concentrations in serum before and after lipoprotein apheresis treatment

The institutional review boards of National Cerebral and Cardiovascular Center and Graduate School of Medicine and Faculty of Medicine at The University of Tokyo approved the study protocol. This study was performed based on the Declaration of Helsinki, and all participants provided written informed consent after explanation of the procedures and possible risks in this study. At the National Cerebral and Cardiovascular Center, patients enrolled in this study were prescribed with 2 mg of doxazosin twice a day, 200 mg of ticlopidine twice a day, or 5 mg of amlodipine once a day. Total cholesterol and VLDL/LDL cholesterol concentrations in each patient were obtained by biochemical test at the National Cerebral and Cardiovascular Center. Serum specimens before and after lipoprotein apheresis were collected and subsequently mixed with orlistat at a final concentration of 10 µM. VLDL/LDL particles in serum specimens were separated using LDL/VLDL and HDL Purification Kits (Cell Biolabs, San Diego, CA). Then, separated VLDL/LDL particles were pretreated as described in “Sample preparation for drug quantification”. Drug concentrations in each specimen were quantified by LC/MS/MS and normalized using the recovery rate of VLDL/LDL particles, which was calculated as the ratio of VLDL/LDL cholesterol concentration after kit purification as described above to that determined by biochemical tests at the National Cerebral and Cardiovascular Center Hospital.

### Statistical analysis

Statistical analysis was performed as follows: the two-tailed unpaired Student’s *t* test was used to analyze the results of *in vitro* assays and *in vivo* studies (Figs 2A,C–F, 3, 5 and 6, S3, S4, S6, and S7). Bonferroni’s method was used to analyze the results of *in vitro* assays under the LDLR-knockdown condition and *in vivo* studies (Figs 1G, 2I and 4). The paired *t* test was used to analyze changes in serum drug concentration after lipoprotein apheresis treatment (Fig. 7).

## Electronic supplementary material

Supplementary Materials
