# Supplementary material for: VLDL/LDL acts as a drug carrier and regulates the transport and metabolism of drugs in the body
Source: Sci Rep. 2017 Apr 4;7:633. doi: 10.1038/s41598-017-00685-9 (PMC5428859; doi:10.1038/s41598-017-00685-9)
Supplement: Supplementary file 1 — Supplementary Materials [file 41598_2017_685_MOESM1_ESM.pdf]

Title

VLDL/LDL acts as a drug carrier and regulates the transport and metabolism of drugs in the body

Authors

Hideaki Yamamoto[1†], Tappei Takada[1†*], Yoshihide Yamanashi[1†], Masatsune Ogura[2†], Yusuke Masuo[1], Mariko Harada-Shiba[2] and Hiroshi Suzuki[1*]

Affiliations

[1]Department of Pharmacy, The University of Tokyo Hospital, Faculty of Medicine, The University of Tokyo, Tokyo 113-8655, Japan.

[2]Department of Molecular Innovation in Lipidology, National Cerebral and Cardiovascular Center Research Institute, Osaka 565-8565, Japan

[†]These authors contributed equally to this work.

[*]Corresponding authors. E-mail: tappei-tky@umin.ac.jp (T.T.), suzukihi-tky@umin.ac.jp (H.S.)

Supplementary Materials

Supplementary Tables S1–S2

Supplementary Figures S1–S8

Supplementary Materials

Supplementary Table S1. BDDCS information on the investigated drugs.

Class 1	Class 2	Class 3	Class 4
Acetaminofene	Amiodarone	Atenolol	Enoxacin
Amlodipine	Carbamazepine	Cimetidine	Sulfadiazine
Bupivacaine	Clopidogrel	Clarithromycin	
Chlorpropamide	Glibenclamide	Disopyramide	
Colchicine	Glimepiride	Fluconazole	
Diclofenac	Imatinib	Methotrexate	
Doxazosin	Lovastatin	Methyldopa	
Fluvastatin	Miconazole	Nizatizine	
Imipramine	Simvastatin	Procainamide	
Labetalol	Sorafenib	Rosuvastatin	
Metoprolol	Sulindac		
Metronidazole	Tamoxifen		
Rosiglitazone	Testosterone		
Ticlopidine	Ticagrelor		
Verapamil	Tolbutamide		

Supplementary Table S2. Mass spectrometer and UPLC conditions. The mass spectrometer and UPLC conditions used to determine drug concentrations are shown.

Compound	Retention Time [min]	Detection Condition
**Amiodarone**	3.74	Parent Mass: 645.78<br>Daughter Mass: 99.83
**Amlodipine**	3.21	Parent Mass: 409.40<br>Daughter Mass: 238.04
**Clarithromycin**	3.26	Parent Mass: 748.64<br>Daughter Mass: 158.10
**Clopidogrel**	4.14	Parent Mass: 322.21<br>Daughter Mass: 183.90
**Labetalol**	2.98	Parent Mass: 329.39<br>Daughter Mass: 162.10
**Sulfadiazine**	3.92	Parent Mass: 251.01<br>Daughter Mass: 92.04
**Ticagrelor**	2.83	Parent Mass: 523.26<br>Daughter Mass: 152.91
**Ticlopidine**	3.02	Parent Mass: 264.25<br>Daughter Mass: 89.04
**Tolbutamide**	3.70	Parent Mass: 271.31<br>Daughter Mass: 91.00
**2-oxo clopidogrel**	4.12	Parent Mass: 338.20<br>Daughter Mass: 182.93
**Doxazosin**	2.95	Parent Mass: 452.10<br>Daughter Mass: 344.15
**Pioglitazone (IS)**	3.01	Parent Mass: 357.15<br>Daughter Mass: 134.00

Solvent A: water with 0.1% formic acid
Solvent B: acetonitrile with 0.1% formic acid

Gradient Elution Method

Time [min]	Solvent A [%]	Solvent B [%]
0	100	0
1.0	100	0
3.5	2	98
5.0	2	98
5.1	100	0

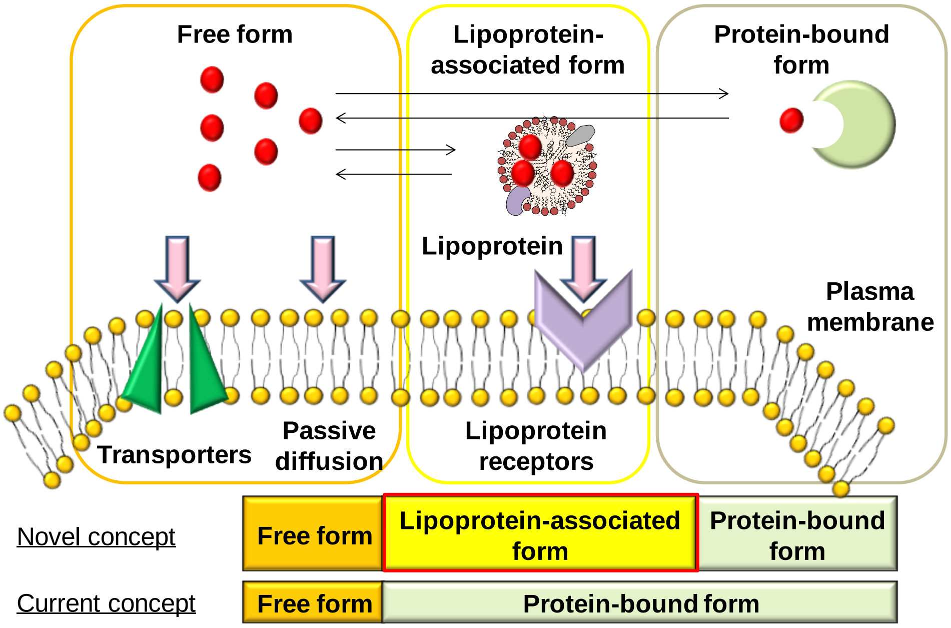

Supplementary Fig. S1. A novel concept in drug behavior: lipoprotein-mediated drug transport. Representation of lipoprotein-mediated drug transport. Drugs in circulation are typically categorized into an unbound free form or a protein-bound form, and only the free form is thought to move into tissues. Based on our present findings, we propose that the lipoprotein-associated form should be distinguished from the protein-bound form because of its ability to transfer into tissues.

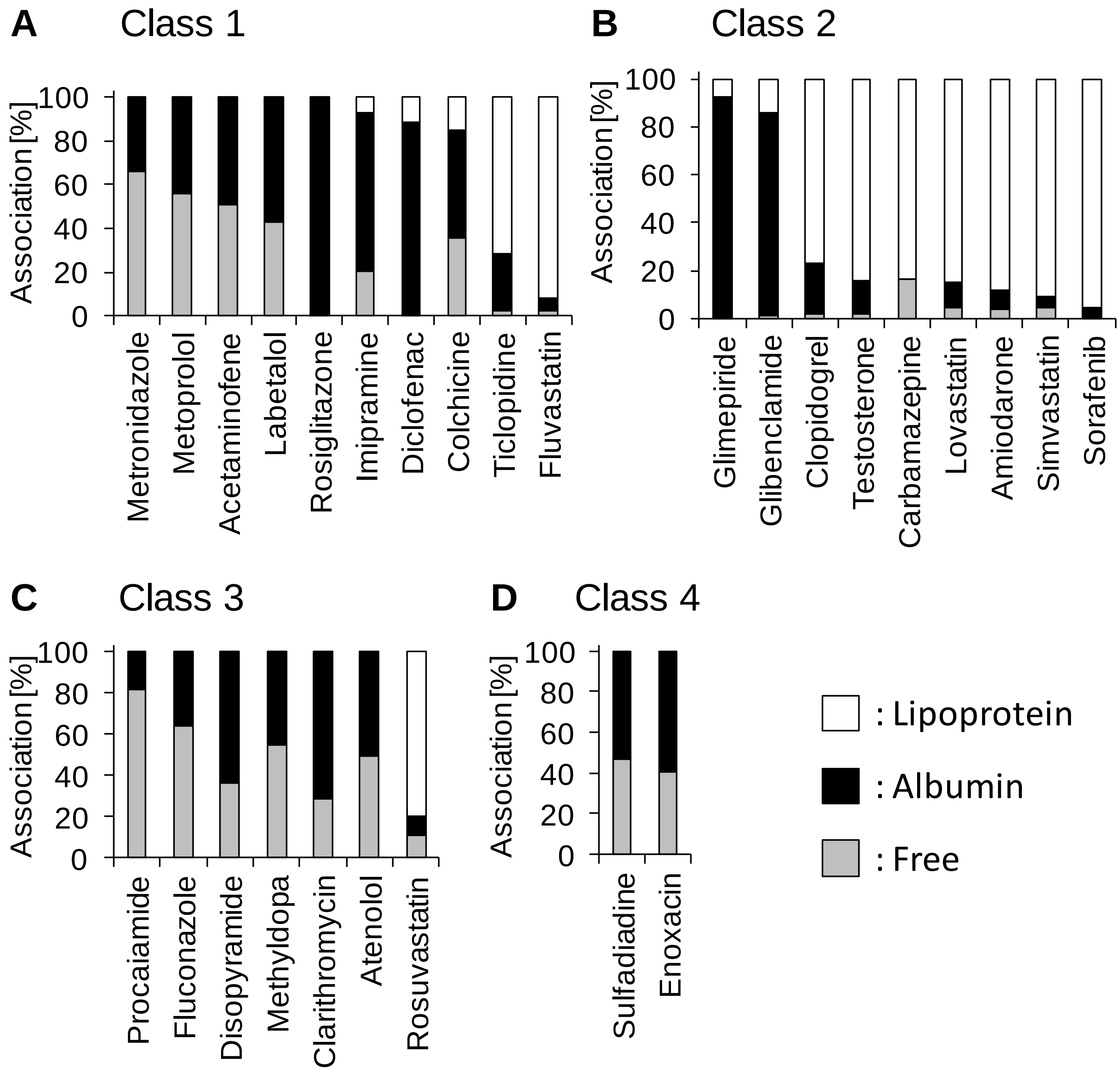

Supplementary Fig. S2. Association of various drugs with lipoproteins. According to the amount of each drug in lipoprotein fractions (white bar), albumin fractions (black bar), and free fractions (gray bar), drug association with each fraction was calculated. **(A)** Class 1, **(B)** Class 2, **(C)** Class 3, and **(D)** Class 4 drugs.

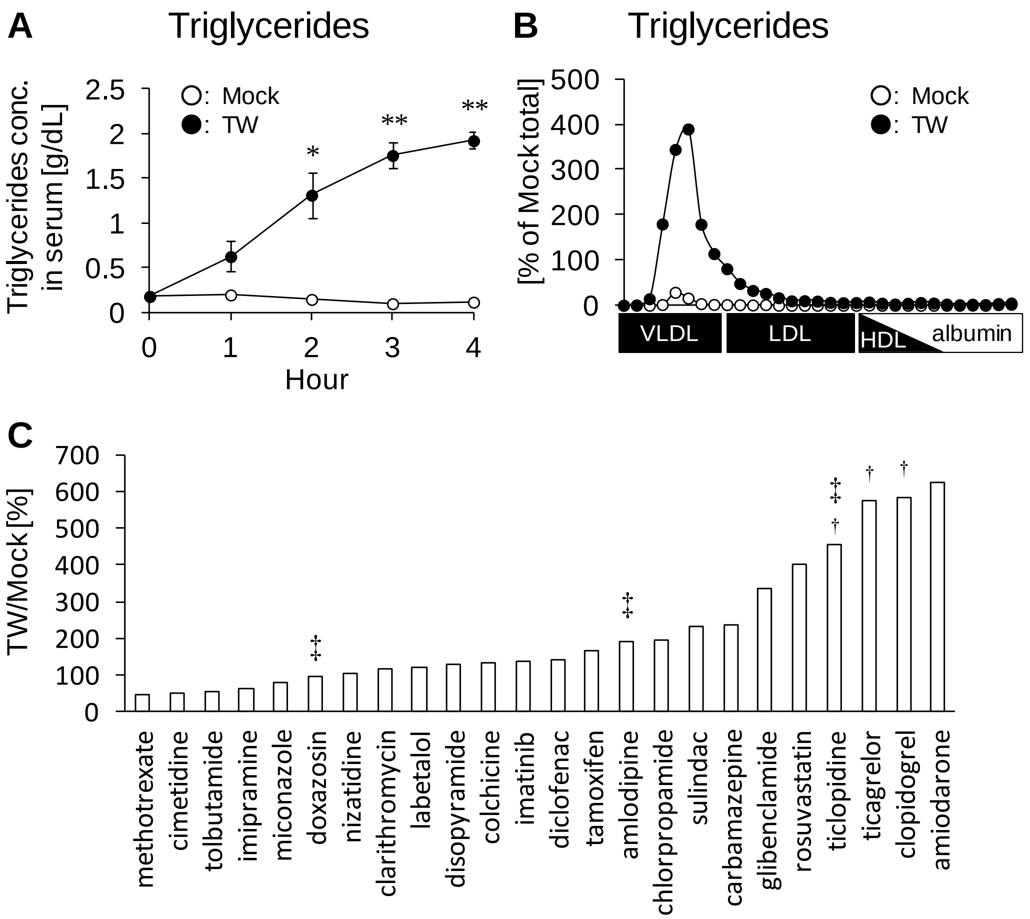

Supplementary Fig. S3. Effect of TW treatment on drug serum concentrations. (A) Serum triglycerides concentrations after intravenous administration of saline (Mock) or Triton WR-1339 (TW). **(B)** Association of triglycerides with lipoproteins at 4 h after TW treatment. **(C)** AUC ratio of each drug in TW-treated mice to that in Mock-treated mice. "†" represents P2Y12 receptor antagonists. "‡" represents analytes for the clinical study. Bars represent the mean ± s.e.m. *p<0.05, **p<0.01.

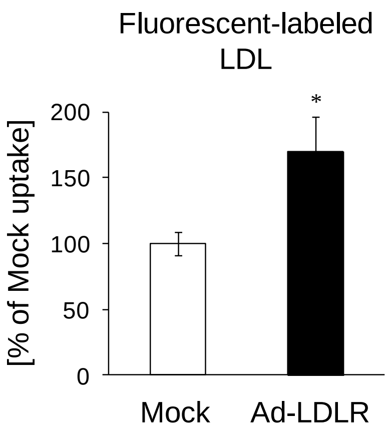

Supplementary Fig. S4. Effect of LDLR overexpression on fluorescent-labeled LDL transport *in vitro*. Hepa1-6 cells were infected with green fluorescent protein (GFP)-tagged mouse LDLR-expressing adenovirus (Ad-LDLR). GFP-expressing adenovirus (Mock) was used as a control. Two days after infection, uptake of fluorescent-labeled LDL in Mock and Ad-LDLR cells was quantified by measuring fluorescence (excitation/emission = 540/570 nm). Bars represent the mean ± s.e.m. *$p<0.05$.

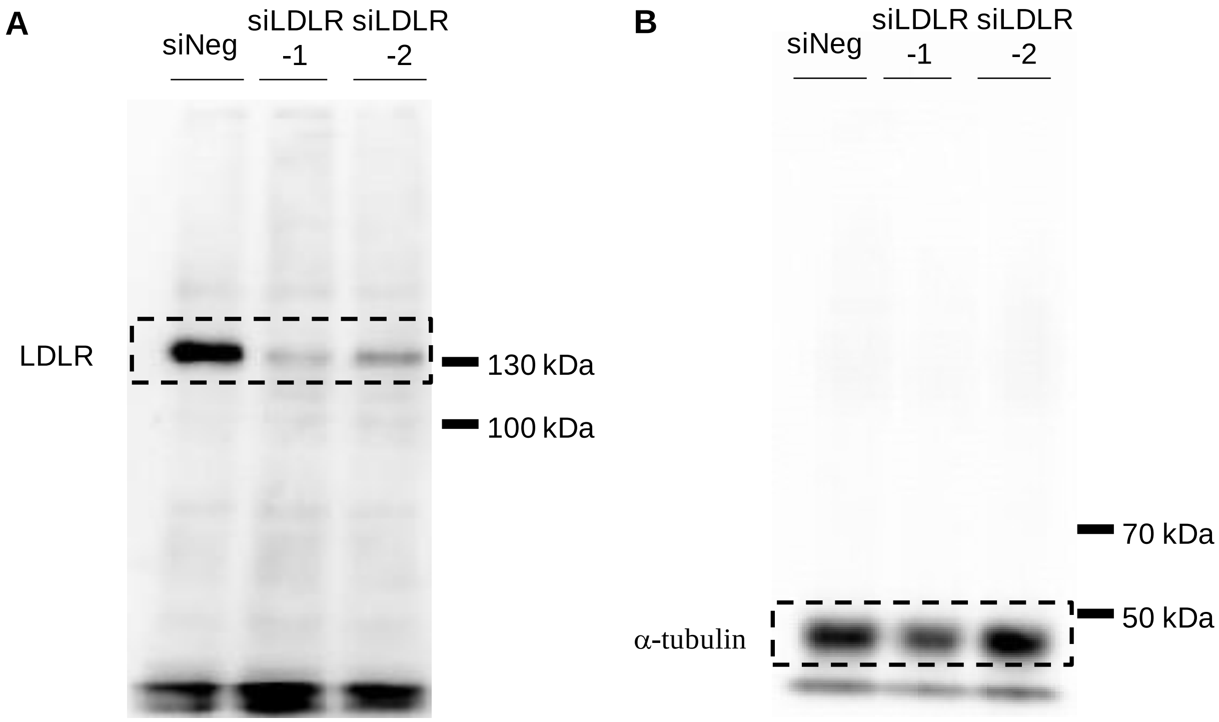

Supplementary Fig. S5. Effect of siRNA against LDLR on the LDLR expression level in Huh-7 cells. Huh-7 cells were transfected with control siRNA (siNeg) or siLDLR (siLDLR-1 and siLDLR-2). Seventy-two hours after transfection, protein levels in whole cell lysates were quantified by western blotting. Representative images of four experiments are shown. **(A)** LDLR, and **(B)** α-tubulin.

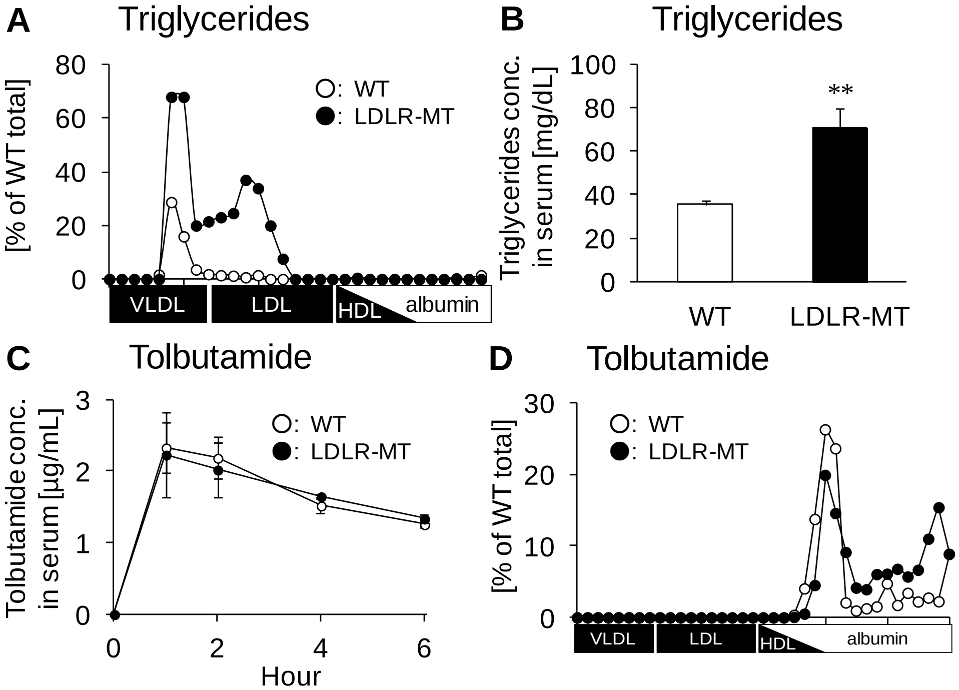

Supplementary Fig. S6. Clarification of VLDL/LDL-mediated drug transport *in vivo*. (A) Association of triglycerides with lipoproteins in wild-type (WT) and LDLR-mutant (LDLR-MT) mice. **(B)** Serum triglycerides concentrations in WT and LDLR-MT mice. **(C)** Serum tolbutamide concentrations in WT and LDLR-MT mice. **(D)** Association of tolbutamide with lipoproteins in WT and LDLR-MT mice at 4 h after clopidogrel administration. Bars represent the mean ± s.e.m. **$p<0.01$.

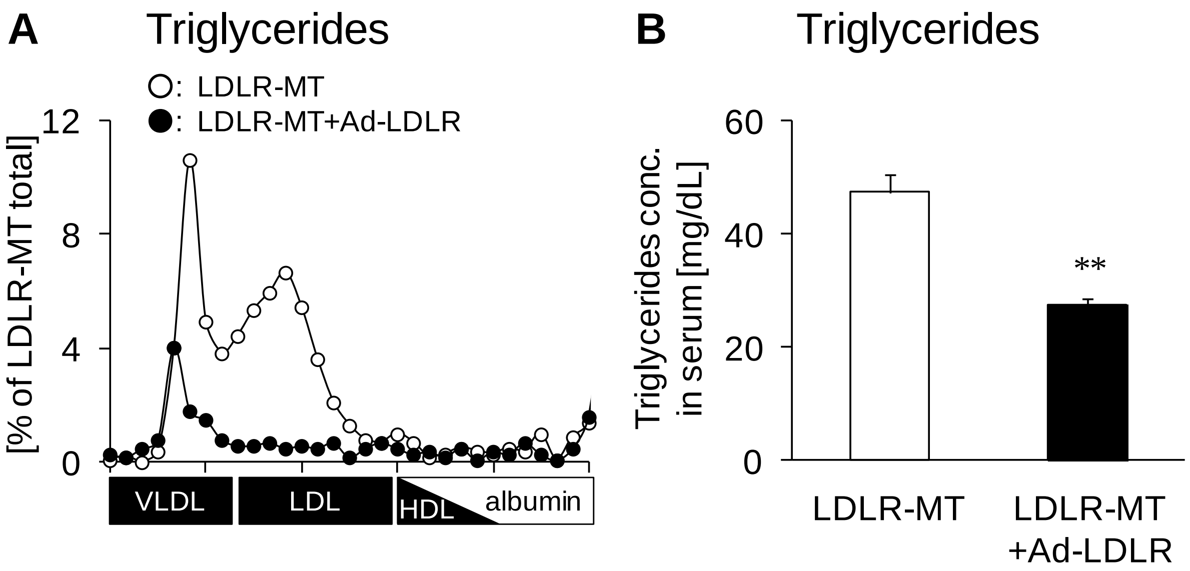

Supplementary Fig. S7. Effect of LDLR overexpression on lipid status *in vivo*. (A) Association of triglycerides with lipoproteins in LDLR-mutant (LDLR-MT) and adenovirus-mediated LDLR-rescued LDLR-MT (LDLR-MT+Ad-LDLR) mice. **(B)** Serum triglycerides concentrations in LDLR-MT and LDLR-MT+Ad-LDLR mice. Bars represent the mean ± s.e.m. **p<0.01.

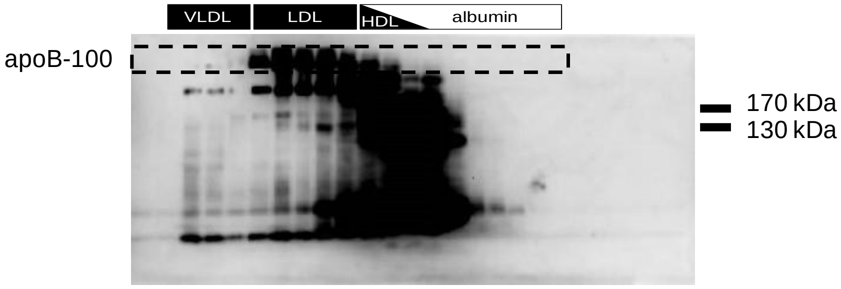

Fig. 1A

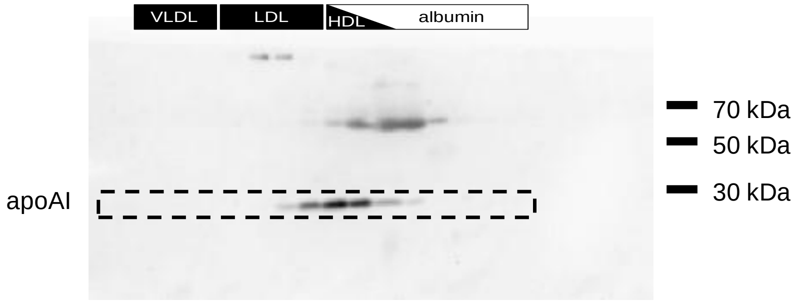

Fig. 1A

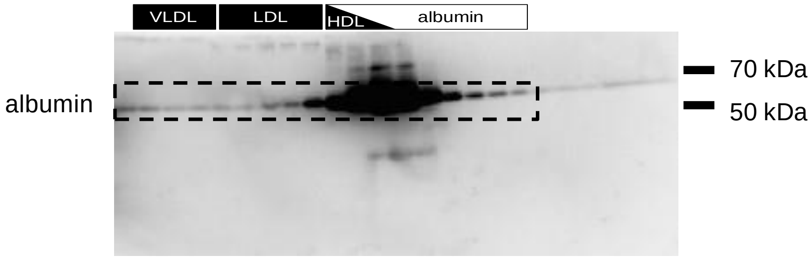

Fig. 1A

Supplementary Fig. S8. (continued below; legend on the next page)

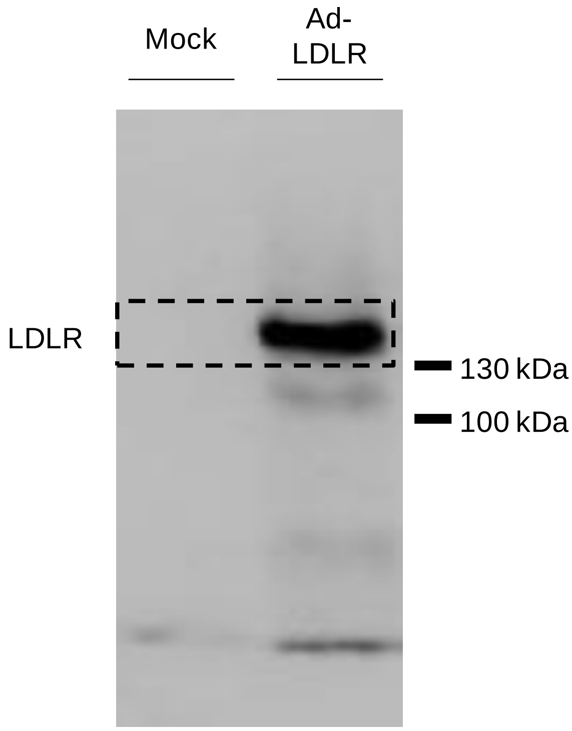

Fig. 3A

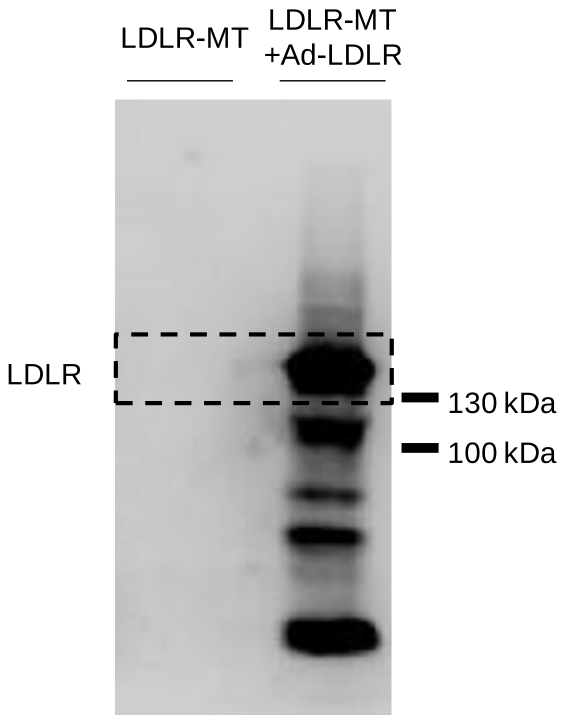

Fig. 6A

Supplementary Fig. S8. Full length images of western blots used in this manuscript. Boxes indicate the approximate portions of the membranes that are included in the figures.
